# *Panax ginseng* Total Protein Facilitates Recovery from Dexamethasone-Induced Muscle Atrophy through the Activation of Glucose Consumption in C2C12 Myotubes

**DOI:** 10.1155/2019/3719643

**Published:** 2019-08-06

**Authors:** Rui Jiang, Manying Wang, Lei Shi, Jingyuan Zhou, Rui Ma, Kai Feng, Xuenan Chen, Xiaohao Xu, Xiangyan Li, Tong Li, Liwei Sun

**Affiliations:** ^1^Jilin Technology Innovation Center for Chinese Medicine Biotechnology, College of Science, Beihua University, Jilin 132013, China; ^2^Research Center of Traditional Chinese Medicine, The Affiliated Hospital to Changchun University of Chinese Medicine, Changchun, Jilin 130021, China; ^3^Departments of Pathology, The Johns Hopkins University School of Medicine, Baltimore, Maryland 21205, USA

## Abstract

**Background:**

The clinical anti-inflammatory drug dexamethasone (DEX) can cause many side effects such as muscle atrophy for long-term use. Muscle atrophy induced by DEX may be caused by decrease of glucose consumption. Panax ginseng C.A. Meyer was previously considered to be an antiatrophic agent for glucocorticoid- (GC-) treated therapies. As one of the main components, it remains unclear whether ginseng total protein (GP) facilitates recovery from muscle atrophy induced by DEX.

**Methods:**

In this study, GP was extracted and purified with Sephadex-G50. C2C12 myoblasts was induced with 2% horse serum to differentiate into C2C12 myotubes. Cell viability was analyzed by the MTT assay, and Ca^2+^ concentration was analyzed by a flow cytometer. The release of lactic dehydrogenase (LDH) and the glucose consumption were analyzed by spectrophotometry. The phosphorylation of AMP-activated protein kinase (AMPK), phosphoinositide 3-kinase (PI3K), and protein kinase B (Akt) and the expression of glucose transporter 4 (GLUT4) were analyzed by Western blotting. The phosphorylation of AS160 was quantified by Immunofluorescence staining.

**Results:**

We found that GP increased cell viability and increased myotube diameter in high-dose DEX-treated C2C12 myotubes for 24 h, but this activity was not found in the enzymatic hydrolyzed GP group. GP reduced muscle atrophy by decreasing the expression of key proteins such as muscle RING-finger protein-1 and muscle atrophy F-box, reducing the Ca^2+^ concentration, and decreasing the release of LDH in DEX-injured C2C12 myotubes. Moreover, GP improved glucose consumption and increased the phosphorylation of AMPK, PI3K, Akt, and AS160 and the expression of GLUT4 in DEX-treated C2C12 myotubes.

**Conclusion:**

The results of this study suggest that GP has effects on recovering DEX-induced muscle atrophy and cell injury, which may improve glucose consumption via the AMPK and PI3K/Akt pathways in high-dose DEX-treated C2C12 myotubes. This study provides* in vitro* mechanistic insights into the recovery of muscle atrophy with GP treatment.

## 1. Introduction

As a synthetic glucocorticoid (GC), dexamethasone (DEX) has anti-inflammatory [1], antiallergic [2], and antishock [3] properties. However, excess DEX leads to defects in glucose metabolism, muscle atrophy, and insulin resistance [4, 5]. Muscle atrophy has profound effects on the daily life of patients, especially on physical activity, and decreases body movements to cause movement disorders and much inconvenience [6]. Therefore, improvement of muscle atrophy caused by DEX is of great significance for expanding its clinical application.

In skeletal muscle, it is well known that high-dose or prolonged GC treatment inhibits glucose consumption and utilization by antagonizing the insulin response, resulting in mitochondrial dysfunction and muscle atrophy [7, 8]. The promotion of glucose transport is mediated by 5'-monophosphate-activated protein kinase (AMPK) [9]. Activation of AMPK enhances the translocation of glucose transporter isoform 4 (GLUT4) to the cell membrane and then increases glucose consumption [10]. Glucose is transported through GLUT4 into skeletal muscle. In addition to involving the AMPK pathway, the insulin receptor-mediated PI3K/Akt pathway participates in regulating GLUT4 expression. The Akt substrate designated AS160 (160 kDa) is a Rab GTPase-activating protein that modulates GLUT4 trafficking in insulin-sensitive L6 myoblasts [11]. Muscle atrophy induced by DEX begins with a decrease in myotube diameter, which is reflected by both a decrease in synthesis and an increase in protein degradation [12]. This process is mainly caused by activating the ubiquitin-proteasome system, which is mediated by key proteins such as atrogin-1/muscle atrophy F-box and muscle RING finger 1 (MuRF 1) [13]. These are two muscle-specific E3 ubiquitin ligases that are expressed early in the muscle atrophy process; their expression precedes the loss of muscle mass.

Mouse skeletal muscle-derived C2C12 myoblasts are immortal mouse skeletal myoblasts originally derived from satellite cells of the thigh muscle [14], and have been used in many studies as an* in vitro* cell atrophy model [15]. Many studies have showed that DEX treated on C2C12 myotubes could show cell atrophy by inducing increased protein degradation and other metabolic changes, which seen in atrophying muscle in experimental animals [16]. Therefore,* in vitro* DEX-induced cell atrophy model is convenient and mature to clarify the effects and mechanisms of active ingredients from medicinal herbs.

*Panax ginseng* C.A. Meyer as a Traditional Chinese Medicine is widely used in clinical applications, as it has immunomodulatory activity in mice and rats [17], anti-hyperglycemic effects in C2C12 myotubes and mice [18], analgesic effects, and suppresses oxidative stress in mice [3]. Ginseng has protective effects on muscle atrophy in rats [19]. Panaxatriol derived from ginseng has protective effects on skeletal muscle mass in diabetic mice [20]. Ginsenoside Rg1 prevents muscle protein degradation by regulating Akt/ mTOR/FoxO signaling in C2C12 myotubes [21], and prevents myotube atrophy through activating the Akt/mTOR pathway [13]. Available evidence has suggested that ginseng plays a role in increasing glucose consumption, and may reduce muscle atrophy in the db/db mice: a model for diabetic dyslipidemia [22]. However, it is unclear whether it can inhibit muscle atrophy by regulating the glucose consumption pathway. As a key ingredient of* Panax ginseng* C.A. Meyer, ginseng total protein (GP) has antifatigue activity in mice [23]. We hypothesized that GP may facilitate recovery of skeletal muscle mass after cell atrophy.

## 2. Materials and Methods

### 2.1. Materials

Mouse skeletal muscle-derived C2C12 myoblasts (GNM26) were purchased from the Chinese Academy of Sciences (Shanghai, China). Fetal bovine serum (FBS) was purchased from Clark Bioscience (Richmond, VA, USA). Horse serum was purchased from GIBCO (Life Technologies, Carlsbad, CA, USA). DEX was purchased from Sigma (St. Louis, MO, USA). The lactic dehydrogenase (LDH) Biochemical Analysis Kit was purchased from Jiancheng Bioengineering Institute (Nanjing, China). Fluo-3/AM and the BCA Protein Assay Kit were purchased from Beyotime Biotechnology (Shanghai, China). Protease/phosphatase inhibitor cocktails were purchased from Roche (Basel, Switzerland). Primary antibodies against myogenin, MuRF 1, atrogin-1, phosphorylated AMPK (p-AMPK), AMPK, phosphorylated PI3K (p-PI3K), PI3K, phosphorylated Akt (p-Akt), Akt, GLUT4, and GAPDH were obtained from Cell Signaling Technology (Beverly, MA, USA). p-AS160 was obtained from OmnimABs (Alhambra, CA, USA). Chemiluminescence reagents were purchased from Santa Cruz Biotechnology (Santa Cruz, CA, USA).

### 2.2. Preparation of GP

Five-year-old ginseng roots were collected from Fusong in the Province of Jilin, China (latitude, 127.27; longitude, 42.33) in September 2016 and were identified by Jie Wu, a ginseng expert of Jilin Province. The voucher specimen (2145) was conserved at the Herbarium of College of Science, Beihua University (Jilin province, China). GP was prepared by the same method in the published article of the same laboratory [24]. Fresh ginseng roots (1 kg) were cut into small slices and extracted twice with phosphate-buffered saline (PBS) at 4°C for 4 h. The supernatant was condensed with an ultrafiltration membrane (30 kDa) and the concentrated solution was separated with Sephadex G50, the purity of which reached 90.2%. The filtrate was dried using a vacuum freeze dryer. GP purity was measured by a BCA Protein Assay Kit, and the molecular weight distribution was detected by sodium dodecyl sulfate polyacrylamide gel electrophoresis (SDS-PAGE). To further test the anti-atrophy effects of GP, we hydrolyzed GP with 50 *μ*g/mL proteinase K for 10 or 30 min, which were inactivated at 85°C for 1 h.

### 2.3. Cell Culture and Differentiation

C2C12 myoblasts were cultivated in high-glucose Dulbecco's modified Eagle's medium (DMEM) containing 25 mM glucose, 10% FBS, 100 units/mL penicillin, and 100 *μ*g/mL streptomycin and were incubated at 37°C in a water-saturated atmosphere of 5% CO_2_. When the myoblasts were 70–80% confluent, C2C12 myoblasts (7.5 × 10^4^ cells/well) were seeded in a 6-well and 5 × 10^4^ cells/well were seeded in a 96-well plate, and induced to fuse into C2C12 myotubes using high-glucose DMEM containing 25 mM glucose and 2% horse serum for 1 to 7 days [13]. We observed the cell morphology by Giemsa staining to delineate the differentiated cell status. Western blotting analysis was used to determine the level of myogenin, a marker of myogenic differentiation.

### 2.4. Treatment with DEX and GP

After 5 d of differentiation, C2C12 myotubes were subdivided into five groups: (1) the control group, in which cells were incubated for 24 h in medium; (2) the DEX group, in which cells were treated with 200 *μ*M DEX for 24 h; and (3) the DEX + GP group, in which cells were treated with DEX plus GP (5, 10, and 20 *μ*g/mL) for 24 h. All groups were harvested for experiments.

### 2.5. Cell Viability Assay

Cell viability was assessed by the MTT assay [25]. Briefly, C2C12 myotubes (5 × 10^4^ cells/well) were seeded in 96-well plate for 24 h, and treated with DEX and/or different concentrations of GP or bovine serum albumin (BSA) for 24 h. After incubation with 0.5 mg/mL MTT for 4 h at 37°C, 150 *μ*L dimethyl sulfoxide (DMSO) was added to the cells and absorbance was measured at 490 nm. Cell viability was expressed as a percentage relative to the untreated control cells or DEX-treated cells.

### 2.6. Measurement of Myotube Diameters

To evaluate the effects of GP on the atrophy of C2C12 myotubes induced by DEX, the cell diameters were measured according to a previously described method with some modifications [15]. Briefly, C2C12 myoblasts (5 × 10^4^ cells/well) were seeded in 96-well plates for 24 h and then treated with DEX and/or different concentrations of GP (5, 10, 20 *μ*g/mL) for 24 h. Myotubes were fixed in 4% paraformaldehyde for 10 min before being stained with 10% Giemsa, and observed by light microscopy. The diameters of C2C12 myotubes were calculated (n = 200) using Gen 5 data analysis software (BioTek, Winooski, VT, USA).

### 2.7. Lactate Dehydrogenase (LDH) Assays

To understand the effects of GP on the extent of cell injury induced by DEX, the release of LDH in the culture supernatant was measured using the LDH Biochemical Analysis Kit. After treatment with GP, 200 *μ*L culture medium from C2C12 myotubes with DEX and/or GP treatment for 24 h was used to examine LDH release by a microplate reader at 450 nm [26].

### 2.8. Measurements of Calcium (*Ca*^2+^) Levels

According to the manufacturer's instructions with some modifications [27], the levels of Ca^2+^ in C2C12 myotubes treated with DEX and/or GP were detected with the Fluo-3/AM calcium indicator. C2C12 myotubes were dissolved in PBS containing 2 mM Fluo 3-AM, and incubated for 30–60 min at 37°C, during which time the Fluo 3-AM was trapped inside the cells by esterase cleavage. Then Ca^2+^ levels were measured by a flow cytometer (BD Biosciences, San Jose, CA, USA).

### 2.9. Glucose Consumption Assay

The glucose consumption assay was performed according to the method of Choi* et al.* [28]. Briefly, after differentiation, the C2C12 myotubes were cultivated overnight and then treated with DEX and/or GP for 24 h. After treatment, the glucose concentration in medium was measured with the sulfuric acid-phenol method. The amount of glucose consumed was calculated by measuring the glucose concentration in cell-plated wells from that in blank wells.

### 2.10. Western Blotting Analysis

Western blotting analysis was performed according to the method of Wang* et al.* [29, 30]. Cells were washed twice with ice-cold PBS, and lysed with RIPA lysis buffer containing protease/phosphatase inhibitor cocktails for 30 min on ice. Equal amounts of protein (30 *μ*g) were loaded and separated on 12% SDS-PAGE gels. After blocking in 5% non-fat milk, membranes were incubated with primary antibodies overnight at 4°C and with horseradish peroxidase-conjugated secondary antibodies for 1 h, after which proteins were visualized using chemiluminescence reagents and the FluorChem HD2 system.

### 2.11. Immunofluorescence Staining

C2C12 cells were fixed in 4% formaldehyde for 15 min, permeabilized with 0.1% Triton X-100, and incubated with phosphorylated AS160 (p-AS160, Thr642) antibodies in microscopy buffer, followed by incubation with fluorescein isothiocyanate-conjugated secondary antibodies. The nucleus was stained with DAPI. Image acquisition and postprocessing were performed with a cell imaging multifunctional test system Cytation 5 (BioTek).

### 2.12. Statistical Analysis

Ordinary one-way ANOVA of variance was used to test differences among the groups. Data are presented as the mean ± SD. GraphPad Prism 7.0 software was applied to all statistical analyses.

## 3. Results

### 3.1. GP Increases Cell Viability of C2C12 Myotubes Injured by DEX

**Figure 1:**
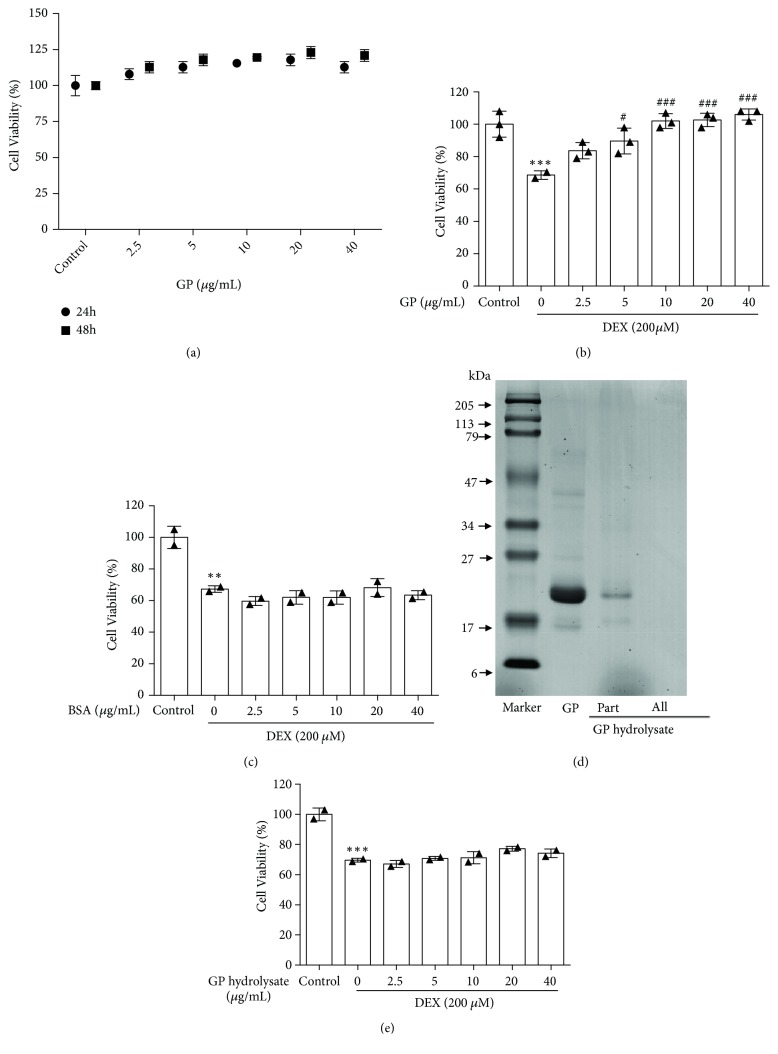
GP increases cell viability induced by DEX in C2C12 myotubes. (a) The MTT assay was used to analyze the cytotoxicity of GP (2.5–40 *μ*g/mL) in C2C12 myotubes after treatment for 24 and 48 h. (b) C2C12 myotubes were treated with GP (2.5–40 *μ*g/mL) for 24 h after incubation with DEX for 24 h. The viability of C2C12 myotubes was assessed by the MTT assay. (c) C2C12 myotubes were treated with BSA (2.5–40 *μ*g/mL) for 24 h after incubation with DEX for 24 h. The viability of C2C12 myotubes was assessed by the MTT assay. (d) SDS-PAGE analysis of GP and GP hydrolysate. (e) C2C12 myotubes were treated with GP hydrolysate (2.5–40 *μ*g/mL) for 24 h after incubation with DEX for 24 h. The viability of C2C12 myotubes was assessed by the MTT assay. Data are expressed as the mean ± SD (n = 3); ^*∗∗*^*p* < 0.01 and ^*∗∗∗*^*p* < 0.001 compared to the control group; ^#^*p *< 0.05 and ^###^*p *< 0.001 compared to the DEX group.

After treatment with different doses of GP (2.5, 5, 10, 20, and 40 *μ*g/mL) for 24 and 48 h, it was shown to have no effect on the viability of C2C12 myotubes (24 h: F=3.11; 48 h: F=11.28, all p>0.05; Figure 1(a)). Based on the muscle-impairing effects of DEX, the effects of GP on skeletal muscle cells injury were assessed. As shown in Figure 1(b), DEX decreased the viability of C2C12 myotubes to 68.61 ± 1.01%, and GP recovered the cell viability of injured C2C12 myotubes induced by DEX in a dose-dependent manner (F=13.67, p<0.05). To determine if all proteins had this effect, we used BSA for functional research analysis, but BSA did not have this effect (F=19.95, all p>0.05, Figure 1(c)), suggesting that not all proteins can increase the cell viability of C2C12 myotubes injured by DEX. To confirm that GP was active, we subjected it to enzymatic hydrolysis. As shown in Figure 1(d), the main molecular weight of GP ranged from 16 to 68 kDa, with the major band present at 22 kDa; this band disappeared after enzymatic hydrolysis with 50 *μ*g/mL proteinase K for 30 min. We also did not find any influence of GP hydrolysate on the viability of C2C12 myotubes (F=32.85, all p>0.05; Figure 1(e)). These results indicated that the effects of promoting C2C12 cell viability were attributed to GP, and that GP reversed DEX-induced injury in muscle myotubes.

### 3.2. GP Decreases DEX-Induced LDH Release and Ca^*2*+^ Concentration Increase in C2C12 Myotubes

**Figure 2:**
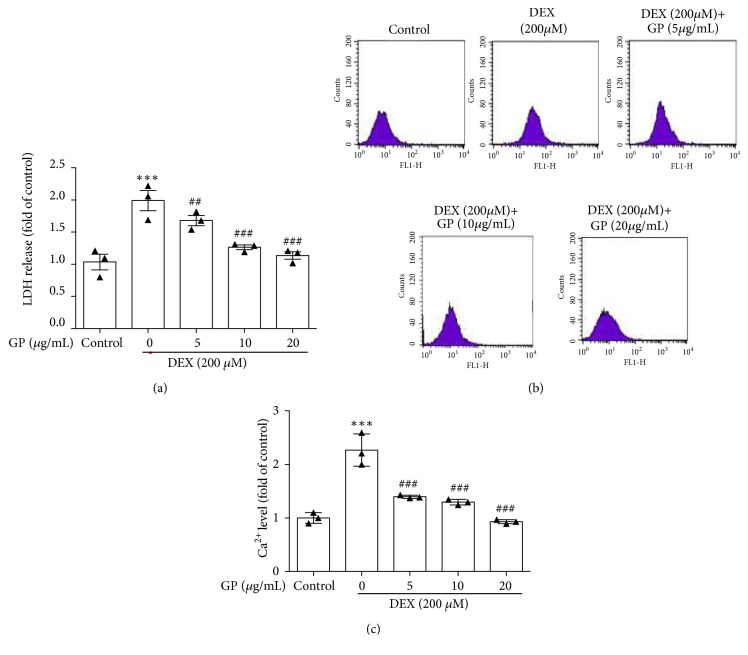
GP decreases DEX-induced LDH release and Ca^2+^ concentration increase in C2C12 myotubes. (a) After treatment with DEX for 24 h, cells were incubated with GP (5–20 *μ*g/mL) for 24 h. LDH release was measured by spectrophotometry. (b) After treatment with DEX for 24 h, cells were incubated with GP (5–20 *μ*g/mL) for 24 h. Ca^2+^ concentration was measured by flow cytometry. (c) Histogram analysis of Ca^2+^ concentration. Data are expressed as the mean ± SD (n = 3); ^*∗∗∗*^*p* < 0.001 compared to the control group; ^##^* p *< 0.01 and ^###^* p *< 0.001 compared to the DEX group.

In this study, the injury of C2C12 myotubes induced by DEX was assessed by measuring LDH release in the culture medium. Compared to control cells, DEX led to a 2.24-fold increase of LDH release, whereas treatment with GP significantly decreased LDH release in DEX-induced C2C12 myotubes in a dose-dependent manner (F=15.81, all p<0.05, Figure 2(a)). Our results showed that the level of intracellular Ca^2+^ was increased up to 2.31-fold with DEX treatment compared to the control. Treatment with 5, 10, and 20 *μ*g/mL GP reduced the concentration of Ca^2+^ by 1.43-, 1.29-, and 0.93-fold, respectively (F=40.65, all p<0.05, Figure 2(c)). These data showed that GP reduced the release of LDH and the concentration of Ca^2+^ induced by DEX in skeletal muscle myotubes.

### 3.3. GP Reduces DEX-Induced C2C12 Myotube Atrophy

**Figure 3:**
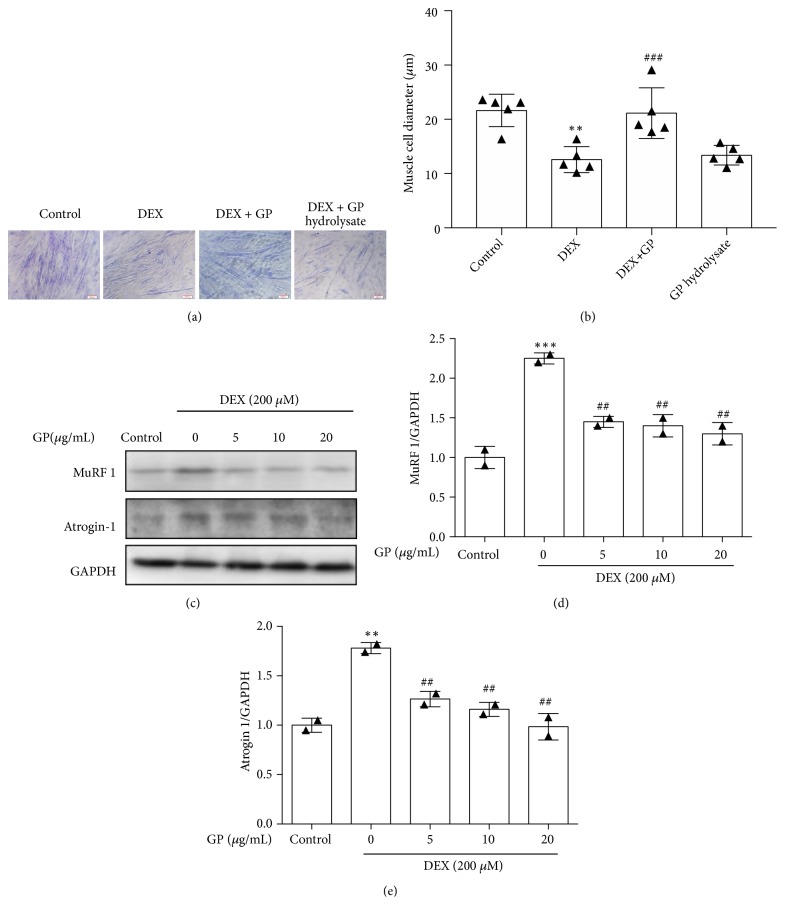
GP reduces DEX-induced C2C12 myotube atrophy. (a) Representative photographs of C2C12 myotubes for the control, DEX, DEX+GP hydrolysate treatments. (b) Comparison of the cell diameters among the four groups measured after completion of the experiments. (c) After treatment with GP for 24 h, the levels of MuRF1 and atrogin1 in DEX-injured C2C12 myotubes were detected by Western blot analysis. (d) The relative expression of MuRF1 was quantified by densitometry analyses. (e) The relative expression of atrogin-1 was quantified by densitometry analyses. GAPDH was used as the loading control. Data are expressed as the mean ± SD (n = 3); ^*∗∗*^*p* < 0.01 and ^*∗∗∗*^*p* < 0.001 compared to the control group; ^##^*p *< 0.01 and ^###^*p*<0.001 compared to the DEX group.

To analyze the effects of GP on the protection of DEX-induced atrophy in myotubes, we first examined myotube diameter. Figures 3(a) and 3(b) shows representative photos of C2C12 myotubes taken immediately after completion of the experiment. Compared to the control group (22.81 ± 0.55 *μ*m), the myotube diameter was significantly decreased upon exposure to 200 *μ*M DEX for 24 h (F=13.24, p<0.001, 14.74 ± 0.48 *μ*m). The myotube size increased after treatment with GP (F=13.24, p<0.001, 22.92 ± 0.58 *μ*m), whereas GP hydrolysate did not increase myotube size (F=13.24, p>0.05, 15.92 ± 0.58 *μ*m). These results indicate that GP plays a role in inhibiting cell atrophy caused by DEX. To further analyze the effects of GP on skeletal muscle cell atrophy, we examined the expression of MuRF 1 and atrogin-1, major atrophy-related E3 ubiquitin ligases specifically expressed in striated muscle. We found that DEX increased the expression of MuRF1 (F=30.82, all p<0.05, Figure 3(d)) and atrogin-1 (F=28.23, all p<0.05, Figure 3(e)) in myotubes. These results showed that GP treatment decreased the expression of MuRF1 and atrogin-1 in DEX-treated myotubes.

### 3.4. GP Increases Glucose Consumption via the AMPK and PI3K/Akt Pathways in DEX-Induced C2C12 Myotube Atrophy

**Figure 4:**
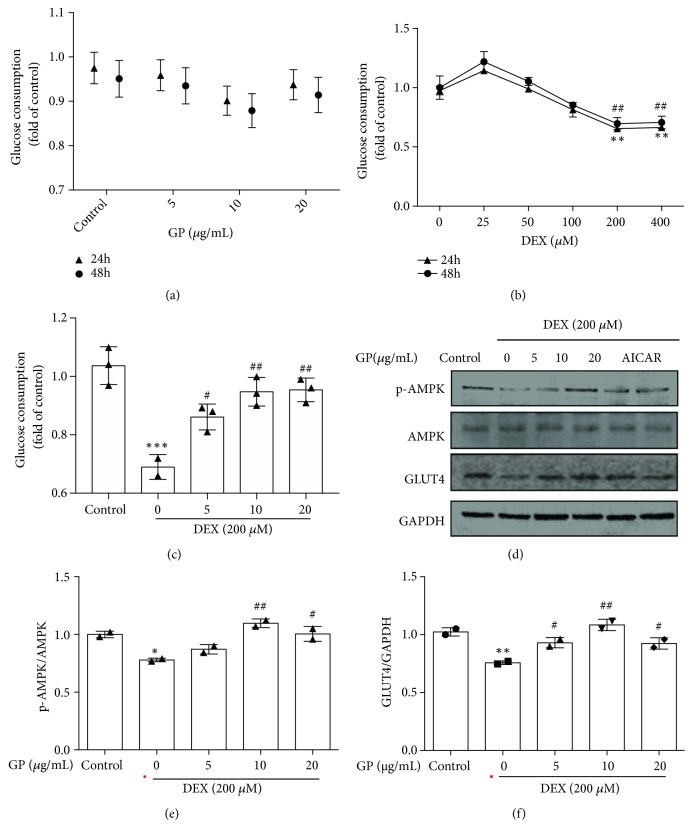
GP increases glucose consumption in DEX-induced C2C12 myotube atrophy by AMPK/GLUT4 pathway. (a) After treatment with GP for 24 and 48 h, glucose uptake was measured by spectrophotometry. (b) After treatment with DEX for 24 and 48 h, glucose uptake was measured by spectrophotometry. ^*∗∗*^*p* < 0.01 versus 24 h; ^##^* p *< 0.01 vs. 48 h. (c) After treatment with DEX for 24 h, cells were incubated with GP (5–20 *μ*g/mL) for 24 h. Glucose uptake was measured by spectrophotometry. (d) After treatment of GP for 24 h, the levels of p-AMPK/AMPK and GLUT4 in DEX-injured C2C12 myotubes were detected by Western blot analysis. (e) The relative expression of p-AMPK/AMPK was quantified by densitometry analyses. (f) The relative expression of GLUT4 was quantified by densitometry analyses. Data are expressed as the mean ± SD (n = 3); ^*∗*^*p* < 0.05, ^*∗∗*^*p* < 0.01, and ^*∗∗∗*^*p* < 0.001 compared to the control group; ^#^* p *< 0.05 and ^##^* p *< 0.01 compared to the DEX group.

**Figure 5:**
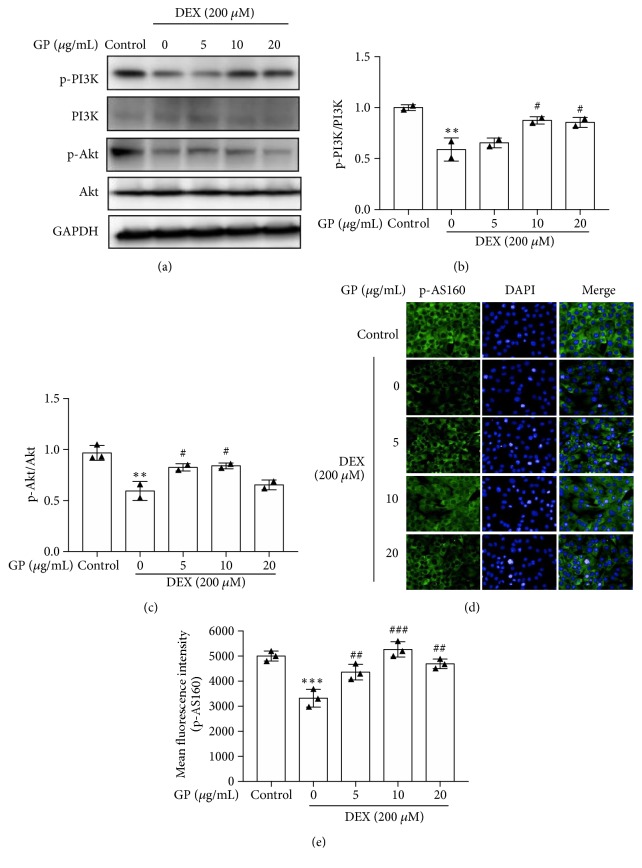
GP increases glucose consumption in DEX-induced C2C12 myotube atrophy by PI3K/Akt pathway. (a) After treatment with GP for 24 h, the levels of p-PI3K/PI3K and p-Akt/Akt in DEX-injured C2C12 myotubes were detected by Western blot analysis. (b) The relative expression of p-PI3K/PI3K was quantified by densitometry analyses. (c) The relative expression of p-Akt/Akt was quantified by densitometry analyses. (d) The relative phosphorylation of AS160 was quantified by Immunofluorescence staining. (e) The relative phosphorylation of AS160 was quantified by densitometry analyses. Data are expressed as the mean ± SD (n = 3);^*∗∗*^*p* < 0.01 and ^*∗∗∗*^*p* < 0.001 compared to the control group; ^#^* p *< 0.05, ^##^* p *< 0.01, and ^###^* p *< 0.001 compared to the DEX group.

As shown in Figure 4(a), GP had no effects on glucose consumption of C2C12 myotubes (24 h: F=1.729, 48 h: F=1.201; all p>0.05). C2C12 myotubes treated with DEX decreased glucose consumption at a dose of 200 *μΜ*. DEX reduced glucose consumption by about 34.75 ± 0.96% compared to the control (Figure 4(b)). Compared to the DEX-treated group, treatment with 5, 10, and 20 *μ*g/mL GP increased glucose consumption by 19.27%, 32.27%, and 29.93%, respectively, (F=16.36, all p<0.05, Figure 4(c)), suggesting that GP increased glucose consumption in DEX-induced C2C12 myotube atrophy. We found that DEX decreased the phosphorylation of AMPK and GLUT4 expression in myotubes. GP increased the phosphorylation of AMPK (F=20.31, all p<0.05; Figure 4(e)) and the expression of GLUT4 (F=18.58, all p<0.05; Figure 4(f)) in DEX-induced C2C12 myotube atrophy, similar to the AMPK activator, AICAR [31]. We also found that DEX decreased the phosphorylation of PI3K and Akt in myotubes. GP restored the phosphorylation of PI3K (F=14.39, all p<0.05; Figure 5(b)) and Akt (F=13.71, all p<0.05; Figure 5(c)) in DEX-induced C2C12 myotube atrophy. In addition, DEX decreased the phosphorylation of AS160 in myotubes, while GP restored the phosphorylation of AS160 in DEX-induced C2C12 myotube atrophy (F=25.46, all p<0.05; Figure 5(e)). These results indicated that GP reduced DEX-induced C2C12 myotube atrophy by enhancing the AMPK and PI3K/Akt pathways, which play a role in glucose consumption in skeletal muscle cells.

## 4. Discussion

GC has a wide range of clinical applications, but can cause many myopathies. High-dose synthetic GC DEX can induce muscle fibers to become thinner and decrease muscle mass. Muscle atrophy caused by DEX is caused by an increase in muscle protein degradation and a decrease of muscle protein synthesis. This phenomenon may occur by activating catabolic signals including ubiquitin E3 ligases MuRF1 and atrogin-1 [32]. Fructus Schisandrae [33], celastrol [15], ginsenoside Rb1 [21], and many Traditional Chinese Medicines [34] or extracts [35] have antiatrophy effects, even when used in the clinic. GP also had similar effects, and as such, has potential therapeutic applications. Our results showed that GP increased cell viability from DEX-induced cell injury and protected the injured cell membrane by decreasing the level of LDH release. GP had protective effects by increasing the release of Ca^2+^ accumulation to reduce injury from DEX in C2C12 myotubes. Our results also showed that myotube size and the expression of MuRF 1 and atrogin-1 were increased after treatment with GP in DEX-treated myotubes. The results showed that GP inhibited C2C12 myotube atrophy induced by DEX by regulating the expression of MuRF 1 and atrogin-1. Muscle tissue is composed of many muscle fibers, and myotubes are basic units that make up the muscle fibers. Therefore, myotube atrophy recovery by GP may provide an experimental basis for using this protein to recover muscle atrophy* in vivo*.

Skeletal muscle is one of the major tissues responsible for glucose homeostasis, as about 80% of glucose utilization occurs in skeletal muscle [36]. Some evidence has shown that GCs have very complex effects on glucose consumption in various tissues and cells. It has also been shown that GCs can inhibit glucose consumption and induce muscle cell atrophy [15]. In accordance with these reports, we found that treatment with DEX decreased glucose consumption in C2C12 myotubes, which was attenuated by GP. These results indicate that GP might partially protect C2C12 myotubes against DEX-induced atrophy by improving the glucose consumption of these cells. Ginsenoside Rg1 prevents myotube atrophy by activating the Akt/mTOR/FoxO target pathway [21]. Thus, GP can recover DEX-induced muscle atrophy by regulating glucose consumption, which differs from the reported pathway of Ginsenoside Rg1.

Glucose is transported through GLUT4 into skeletal muscle. In addition to the AMPK pathway, the insulin receptor-mediated PI3K/Akt pathway also participates in the regulation of GLUT4 expression [10, 37]. AMPK is a master sensor and regulator, which plays a role in cellular energy homeostasis and mediates the contraction-evoked promotion in glucose transportation [38]. Glucose is transported through GLUT4 into skeletal muscle. AS160 is a molecular link among diverse signaling cascades converging on GLUT4 translocation. The activation of PI3K/AKT results in phosphorylation of the Akt substrate AS160 to induce the cellular distribution of GLUT4. In turn, the activity of PI3K recruits the serine/threonine kinase Akt to the cell membrane. This feedback loop is thought to be critical for insulin-mediated GLUT4 translocation from endosomal storage sites to the sarcolemma. Our results showed that GP led to a dose-dependent increase in the phosphorylation of AMPK, PI3K/Akt, and AS160 and increased GLUT4 expression to increase glucose transport in injured skeletal muscle cells. Based on these findings, GP can ameliorate injury from muscle contractions by increasing glucose consumption via the AMPK and PI3K/Akt signaling pathways. In addition, AMPK can trigger autophagy through various mechanisms such as the ULK1-mTOR signaling pathway. Further investigation of the effects of GP on autophagy will aid in providing an understanding of its protective effects on injured skeletal muscle cells.

To the best of our knowledge, this provides mechanistic insights into the protective effects on muscle atrophy from GC-induced atrophy in muscle myotubes. The results demonstrate that GP facilitates recovery from high-dose DEX-induced muscle atrophy through the activation of glucose consumption* in vitro*. Confirmation of these results by* in vivo* studies is needed.

## Data Availability

The data used to support the findings of this study are available from the corresponding author upon request.
